# Extracellular Vesicles Obtained from Hypoxic Mesenchymal Stromal Cells Induce Neurological Recovery, Anti-inflammation, and Brain Remodeling After Distal Middle Cerebral Artery Occlusion in Rats

**DOI:** 10.1007/s12975-024-01266-5

**Published:** 2024-09-07

**Authors:** Mihaela Abuzan, Roxana Surugiu, Chen Wang, Ayan Mohamud-Yusuf, Tobias Tertel, Bogdan Catalin, Thorsten R. Doeppner, Bernd Giebel, Dirk M. Hermann, Aurel Popa-Wagner

**Affiliations:** 1 Department of Neurology, University Hospital Essen, University of Duisburg-Essen, Essen, Germany; 2 Experimental Research Center in Normal and Pathological Aging (ARES), University of Medicine and Pharmacy, Craiova, Romania; 3 Institute for Transfusion Medicine, University Hospital Essen, University of Duisburg-Essen, Essen, Germany; 4 Department of Neurology, University Hospital Gießen and Marburg, Campus Gießen, Giessen, Germany

**Keywords:** Angiogenesis, Exosome, Hypoxic preconditioning, Ischemic stroke, Macrophage, Permanent focal cerebral ischemia

## Abstract

Small extracellular vesicles (sEVs) obtained from mesenchymal stromal cells (MSCs) have shown considerable promise as restorative stroke treatment. In a head-to-head comparison in mice exposed to transient proximal middle cerebral artery occlusion (MCAO), sEVs obtained from MSCs cultured under hypoxic conditions particularly potently enhanced long-term brain tissue survival, microvascular integrity, and angiogenesis. These observations suggest that hypoxic preconditioning might represent the strategy of choice for harvesting MSC-sEVs for clinical stroke trials. To test the efficacy of hypoxic MSCs in a second stroke model in an additional species, we now exposed 6–8-month-old Sprague–Dawley rats to permanent distal MCAO and intravenously administered vehicle, platelet sEVs, or sEVs obtained from hypoxic MSCs (1% O_2_; 2 × 10^6^ or 2 × 10^7^ cell equivalents/kg) at 24 h, 3, 7, and 14 days post-MCAO. Over 28 days, motor-coordination recovery was evaluated by rotating pole and cylinder tests. Ischemic injury, brain inflammatory responses, and peri-infarct angiogenesis were assessed by infarct volumetry and immunohistochemistry. sEVs obtained from hypoxic MSCs did not influence infarct volume in this permanent MCAO model, but promoted motor-coordination recovery over 28 days at both sEV doses. Ischemic injury was associated with brain ED1^+^ macrophage infiltrates and Iba1^+^ microglia accumulation in the peri-infarct cortex of vehicle-treated rats. Hypoxic MSC-sEVs reduced brain macrophage infiltrates and microglia accumulation in the peri-infarct cortex. In vehicle-treated rats, CD31^+^/BrdU^+^ proliferating endothelial cells were found in the peri-infarct cortex. Hypoxic MSC-sEVs increased the number of CD31^+^/BrdU^+^ proliferating endothelial cells. Our results provide evidence that hypoxic MSC-derived sEVs potently enhance neurological recovery, reduce neuroinflammation. and increase angiogenesis in rat permanent distal MCAO.

**Supplementary Information:**

The online version contains supplementary material available at 10.1007/s12975-024-01266-5.

## Introduction

Following recent advances in recanalization therapies (that is, thrombolysis and/or thrombectomy), considerable efforts are currently made to enhance neurological recovery and brain remodeling in the post-acute stroke phase by restorative treatments. Mesenchymal stromal cells (MSCs) are pluripotent cells that can differentiate into a variety of cell types, including osteoblasts, chondrocytes, myocytes, and adipocytes [18]. Similar to MSCs, small extracellular vesicles (sEVs) derived from MSCs have shown considerable promise as a restorative treatment [10, 13]. Ranging from a size of 50 to 150 nm, sEVs play a vital role in intercellular communication [24]. MSC-derived sEVs carry complex signal cargos that effectively modify disease processes [9, 10].

In a head-to-head study, our group has previously demonstrated that when administered 24 h after transient proximal middle cerebral artery occlusion (MCAO) in mice, MSC-derived sEVs very similarly enhanced motor-coordination recovery, long-term neuronal survival, peri-infarct angiogenesis, and neurogenesis as their parental MSCs [3]. These results were consistent in subsequent studies after permanent distal MCAO in young and old rats by our group, in which we showed that sEV delivery improved motor-coordination recovery and reduced neuroinflammation [4].

In comparison to cellular therapies, extracellular vesicle (EV)-based therapies offer several advantages, such as lack of self-replication and malignant transformation [13]. EVs are easy to handle, and can effectively be sterilized through filtration. Although their biological activity can be predicted more accurately than that of cellular therapeutics, EVs have limited ability to sense environmental changes. Preconditioning of MSCs by exposure to a hypoxic environment is a possible strategy to enhance the functional characteristics of MSCs [6].

In mice exposed to transient proximal MCAO, we previously found that sEVs obtained from MSCs cultured under normoxic (21% O_2_) and hypoxic (1% O_2_) conditions similarly reduced infarct volume and brain leukocyte infiltrates in the acute stroke phase [22, 23]. In contrast to sEVs obtained under normoxic conditions, sEVs obtained under hypoxic conditions also increased blood–brain barrier integrity and promoted peri-infarct angiogenesis, which resulted in a better brain tissue preservation with enhanced neuronal survival and reduced brain atrophy in the chronic phase [7, 23]. Based on these findings, hypoxic conditioning might represent the strategy of choice for harvesting MSC-sEVs for clinical stroke trials.

In line with the Stroke Therapy Academic Industry Roundtable (STAIR) [20], which requests that the therapeutic efficacy of new treatments is demonstrated in at least two stroke models and two species, we here exposed adult Sprague–Dawley rats to permanent distal MCAO and examined the effects of MSC-derived sEVs harvested under hypoxic conditions (1% O_2_) on neurological recovery, ischemic brain injury, brain inflammatory responses, and peri-infarct angiogenesis. Our data confirm a recovery-promoting and restorative action of hypoxia-preconditioned MSC-sEVs in this rat permanent focal cerebral ischemia model.

## Materials and Methods

### Ethics Approval

The experiments conducted at the University of Medicine and Pharmacy Craiova received approval from the Institutional Animal Care and Use Committee (#112–15-11–2017). The experiments were carried out in accordance with national regulations in accordance with the EU Directive 2010/63/EU on the care and use of laboratory animals. Randomization of experiments was strictly implemented following the ARRIVE criteria to ensure unbiased data analysis, including behavioral tests, while the examiners involved were kept completely blinded to the experimental conditions. The authors will provide supporting data upon reasonable request to support the conclusions derived from this study.

### Statistical Planning

Statistical planning was done by a sample size calculator (https://homepage.univie.ac.at/robin.ristl/samplesize.php). Assuming an alpha error of 5% and a beta error (1—statistical power) of 20%, sample size calculation determined that 10 animals were needed per group for behavioral and histochemical analyses, provided that sEVs modified the mean value by 35% and that the standard deviation of the data sample was 25% of the mean value (effect size 1.4).

### Animals

A total of 52 male Sprague–Dawley rats (6–8-month-old) were included in the study. The rats were bred and raised in the animal facility at the University of Medicine and Pharmacy Craiova. Animal weights ranged from 310 to 400 g. The rats were housed in a controlled environment with a standard 12-h/12-h light/dark cycle (lights on from 07:00 to 19:00 h). They had unrestricted access to food and water post-MCAO and were kept at an ambient temperature of 22 °C and a humidity of 40–60%.

### Expansion and Characterization of MSCs

As reported previously, human MSCs were cultivated from bone marrow samples obtained from a healthy donor providing informed consent (source 41.5) [23]. The bone marrow sample was obtained through the bone marrow transplantation center at the University Hospital Essen in collaboration with the Westdeutsche Spender Zentrale (https://www.wsze.de/startseite/index.php). Based on local Ethics Committee permission, bone marrow aliquots can be used for research purposes. The aliquots obtained were anonymized. Specific information regarding the donor’s age or sex was not provided. Hence, donors were not specifically enrolled for this study. MSCs were expanded in low glucose Dulbecco’s modified Eagle medium (DMEM) (Lonza, Basle, Switzerland), supplemented with 10% human platelet lysate (hPL), 100 U/ml penicillin–streptomycin-L-glutamine, and 5 IU/ml heparin (Heparin-Natrium-25000, Ratiopharm, Ulm, Germany). The cells were cultured until they reached approximately 80% confluency and were then passaged. As mentioned earlier [23], the MSCs were characterized in passage 3 following criteria of the International Society of Cell and Gene Therapy (ISCT) [18]. These cells were previously shown to express MSC markers and exhibit the capacity to differentiate into osteogenic and adipogenic lineages [3].

### Hypoxic Preconditioning of MSCs and sEV Harvesting

Starting from passage 3, conditioned medium was collected from MSCs cultured under hypoxic conditions (1% O_2_), as described previously [7]. Hypoxic preconditioning was employed to simulate the impact of hypoxia associated with stroke. MSCs did not reveal any histological evidence of structural damage under hypoxic conditions. Additionally, hypoxia had no effect on MSC viability, as assessed by the 3-(4,5-dimethylthiazol-2-yl)-2,5-diphenyltetrazolium bromide (MTT) assay. To eliminate cell debris, the conditioned media were subjected to centrifugation at 2000 g for 15 min. Subsequently, they were stored at 20 °C until further use. Only conditioned media that tested negative for mycoplasma contamination were utilized.

### Preparation of MSC-sEVs

The conditioned medium was simultaneously thawed and treated. Supernatants were subjected to centrifugation at 6800 g using an Avanti centrifuge (JS-5.3 rotor; k-factor: 7,730; Beckman-Coulter) for 45 min to eliminate cell debris and larger vesicles. Subsequently, sEVs were concentrated using polyethylene glycol 6000 (PEG) precipitation, as described previously [12]. The sEVs were then further precipitated through ultracentrifugation at 110,000 g for 130 min (Ti45 rotor, k-factor: 133) after being washed with 0.9% NaCl solution. MSC-sEV samples were resuspended in 10 mM Hepes/0.9% NaCl (Thermo Fisher Scientific) at a concentration of 4 × 10^7^ cell equivalents per ml (1 U) and stored at − 80 °C. As additional control condition, sEVs were also obtained from MSC culture medium (DMEM low, Lonza) supplemented with 10% platelet lysate (sEV_platelet_).

### Characterization of MSC-sEVs

The characterization of MSC-sEV preparations followed the recommendations provided by the International Society for Extracellular Vesicles (ISEV) [21]. Particle concentration and size were assessed using nanoparticle tracking analysis (NTA) with a Particle Metrix instrument (Meerbusch, Germany), as described previously [19]. The protein concentration was measured using a standardized bicinchoninic acid (BCA) assay with Pierce reagents (Rockford, IL, U.S.A.). Supplementary Table 1 presents the particle concentration, size, protein concentration, and purity of the sEV preparations. Previous studies utilizing Western blots demonstrated the absence of cytosolic markers calnexin and prohibitin, while the presence of exosome markers CD9, CD63, CD81, and syntenin was confirmed in the sEV samples [21]. Additionally, transmission electron microscopy was employed to observe the characteristic shape and size of exosomes within the sEV preparations [21]. We furthermore employed imaging flow cytometry using the AMNIS ImageStreamX Mark II Flow Cytometer (Luminex, Seattle, WA, USA) to confirm the presence of CD9^+^, CD63^+^, and CD81^+^ vesicles in the sEV samples (Supplementary Table 2). The gating strategy applied for the analysis of sEVs is depicted in Supplementary Fig. 1. The antibodies utilized for flow cytometry analysis are listed in Supplementary Table 3.

### Behavioral Tests

The animal handling was conducted by two individuals: one person responsible for the surgical procedure and animal handling and another one person carrying out the behavioral tests. Strict hours were maintained between 9 and 12 a.m. for conducting the behavioral tests. In all animals, tests were done at baseline prior to the surgery and repeated at 3, 7, 14, 21, and 28 days post-MCAO.

- *Rotating pole.* The rotating pole assesses vestibulomotor, visuomotor and sensorimotor function, and limb coordination. The apparatus consists of a horizontal cylinder with a diameter of 12 cm and a length of 160 cm that revolves around its own axis and can be set to rotate at a speed between 0 and 6 revolutions per minute (rpm). To keep the animals from slipping, a semi-hard adhesive film was applied to the cylinder surface, which was cleaned after each usage. The time needed to cross the pole from one end to the other end was determined, while the pole was rotating at 3 rpm or 6 rpm [1].
- *Cylinder test.* The cylinder test measures spontaneous motor-coordination. Using a glass cylinder that measures 20 cm in diameter and 40 cm in height, the asymmetry of forelimb placement was assessed. The number of wall contacts of each forelimb was counted over 5 min, while the animal was exploring the walls. The ratio of (left–right)/(left + right) was used to determine the asymmetry index, where left and right represent the number of wall contacts made by the left and right forelimb, respectively [15].

### Permanent Distal MCAO

The animals were food-deprived prior to surgery for 12 h with free access to water. This strategy was chosen, because a high blood glucose level following food ingestion increases brain infarct volume. Animals were anesthetized with 1–1.5% isoflurane in a mixture of 25% O_2_ and 75% N_2_O. Body temperature was maintained at 37 °C by a homeothermic blanket system. A skin incision was made with the use of a stereomicroscope, and the underlying tissue was detached using fine scissors in an effort to prevent bleedings. After temporal fascia sectioning, the right-sided temporal bone was exposed and trephinated without damaging any of the adjacent arteries or nerves. The right-sided middle cerebral artery was gently exposed, lifted with a tungsten hook (Fine Science Tools, Heidelberg, Germany) attached to a micromanipulator (Märzhäuser Precision Micromanipulator Systems, Applied Scientific Instrumentation, Eugene, OR, U.S.A.), and coagulated using a thermal cauterizer (Cauterizer Kit, Fine Science Tools). To avoid thermal injury to the cortical surface, normal saline was administered on the animal’s brain surface. Both common carotid arteries were occluded for 90 min by prepositioned suture loops. Successful middle cerebral artery occlusion was confirmed by a drop of laser Doppler flow to < 20% of the control value. Both common carotid arteries were reopened after 90 min by suture loop removal. Wounds were carefully sutured, and animals were placed back in their home cages. Animals were fed with moistened food pellets for 3 days to avoid food intake difficulties. Of the 52 animals included in the study, 45 survived the MCAO surgery. Three animals did not show behavioral deficits and were excluded from the study. Two animals revealed excessive weight loss after the stroke and had to be excluded and euthanized.

### MSC-sEV Administration

At 24 h, 3 days, 7 days, and 14 days post-MCAO, (i) vehicle, (ii) platelet-derived sEVs (isolated from cell media, dissolved in 0.9% NaCl), or (iii) sEVs obtained from conditioned media of hypoxic MSCs (2 × 10^6^ or 2 × 10^7^ MSC equivalents/kg, dissolved in 0.9% NaCl) were administered through the animal’s tail vein. Dose selection was made based on a previous mouse study [3], in which 2 × 10^7^ MSC equivalents/kg were administered. This dose is 100 times higher than that previously administered by us in a human patient (2 × 10^5^ MSC equivalents/kg) [12]. To gain further insights into dose–response relationships, we also used a tenfold lower dose in this study (2 × 10^6^ MSC equivalents/kg).

### BrdU Labeling

From 8 to 18 days post-MCAO, bromodeoxyuridine (BrdU; 50 mg/kg/day body weight; Sigma) was intraperitoneally administered to label proliferating endothelial cells.

### Animal Sacrifice

**Fig. 1:**
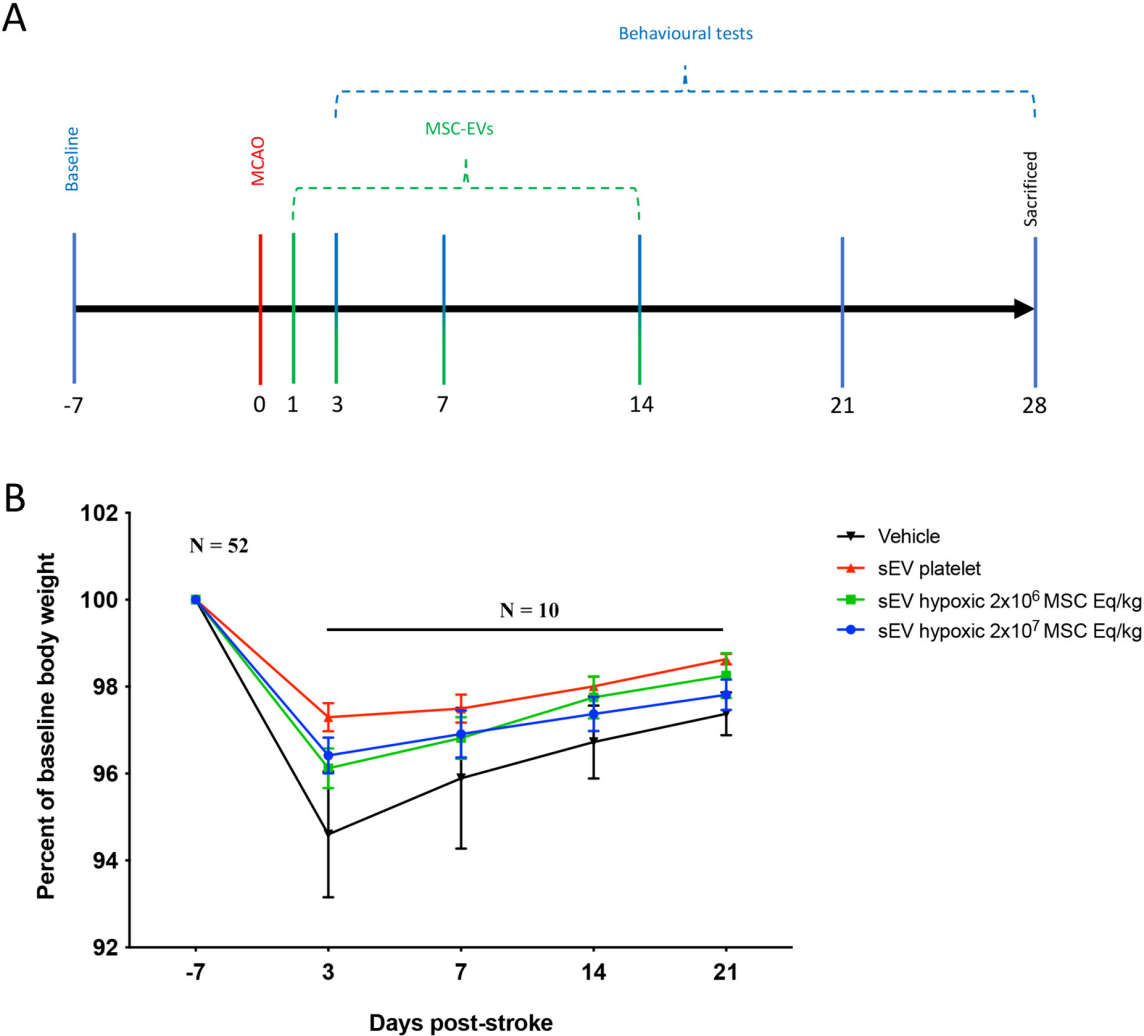
Experimental design and time course of body weight changes in rats subjected to permanent distal middle cerebral artery occlusion (MCAO). A total of 52 animals were included in this study. **A** Temporal sequence of experimental interventions. Rats exposed to permanent distal MCAO received intravenous injections of vehicle, platelet-derived small extracellular vesicles (sEVs), or sEVs obtained from hypoxic (1%O_2_) mesenchymal stromal cells (MSCs; 2 × 10^6^ or 2 × 10^7^ cell equivalents/kg) at four different time points, that is 1, 3, 7, and 14 days after MCAO. Motor-coordination deficits were assessed through behavioral analysis from 3 to 28 days post-MCAO (that is, from 2 to 27 days post-treatment onset). Rats were sacrificed at 28 days post-MCAO for histochemical analysis of brain tissue. **B** Body weight development of rats exposed to permanent distal MCAO (*n* = 10 animals per group). The weight of rats receiving vehicle modestly decreased by ~ 5% subsequent to MCAO. Hypoxic MSC-sEV administration nominally, but not statistically significantly reduced this weight reduction. The body weight of all groups almost completely recovered within 14–21 days post-MCAO. Data did not statistically differ between groups. Data are mean ± standard error of the mean (SEM) values

At 28 days post-MCAO (27 days post-treatment onset), the animals were deeply anesthetized with 2.5% isoflurane in 25% O_2_ and 75% N_2_O, and perfused with 0.1 M phosphate-buffered saline (PBS; pH 7.0) followed by freshly prepared 4% paraformaldehyde in PBS. The brains were removed, post-fixed in 4% paraformaldehyde for 24 h, cryoprotected in 15% glycerol prepared in PBS, flash-frozen in isopentane and kept at − 70 °C until sectioning. Twenty-five-micrometer-thick coronal sections were cut on a freezing microtome. A flow chart of animal experiments is given in Fig. 1A.

### Infarct Volume

For infarct measurement, brain sections obtained at 500-µm intervals (every 20th section) were stained with methyl green/pyronine Y. Infarct areas were measured by an indirect method delineating healthy cortical tissue in both hemispheres using Image J. Partial infarct volumes were calculated using infarct areas at various rostrocaudal levels. Total infarct volume was determined by integrating partial infarct volumes throughout the brain.

### Immunohistochemistry

For histochemical analysis of brain macrophage and microglia accumulation, 25-µm-thick free-floating sections were blocked overnight at 4 °C in PBS containing 3% donkey serum and 0.3% Tween 20 [16, 17]. Sections were incubated for 24 h with monoclonal mouse anti-ED1 antigen (1:500; ab31630, Abcam, Cambridge, U.K.) and polyclonal rabbit anti-Iba1 (1:2000; Wako Chemicals, Neuss, Germany) antibodies. The sections were then exposed to the corresponding goat anti-mouse polymer-HRP or goat anti-rabbit polymer-HRP secondary antibodies (Nichirei Biosciences, Japan). Sections were stained with either with tyramide-FITC or tyramide-Cy3.

For BrdU detection, 25-µm-thick free-floating sections were pre-treated for 2 h at 65 °C with 50% formamide in 0.3 M NaCl containing 10 mM sodium citrate, incubated for 1 h at 40 °C with 2 M HCl before being washed in 0.1 M borate solution (pH 8.5) for 10 min. Sections were incubated for 24 h in monoclonal rat anti-BrdU (1:1000; BU1/75, ab6326, Abcam) and monoclonal mouse anti-CD31 (1:1000; clone MEC13.3, 550274, BD Biosciences, Heidelberg, Germany) antibodies at 4 °C. Fluorescence was detected using secondary goat anti-rat-Cy3 and goat anti-mouse Alexa Fluor 488 antibodies, as adequate.

### Quantitation of ED1^+^ Macrophages and Iba1^+^ Microglia

The number of ED1^+^ activated macrophages and Iba1^+^ microglia was determined in 250 × 250 µm regions of interest using a “random-systematic” protocol (random start point for a systematic series of every 10th section through the infarcted volume) in the Image J software. The area occupied by cells of interest was ~ 30% of the total stained infarct area. In order to prevent false results, only cells with nuclei with a surface area of 70–110 µm^2^ (for activated ED1^+^ macrophages) and 40–70 µm^2^ (for Iba1^+^ microglia) were counted. The surface area of the nuclei of ED1^+^ macrophages was ~ 50% larger than that of monocytes. Hence, monocytes were not included in cell countings. By counting cell caps, cells in the uppermost focal plane were disregarded to avoid oversampling errors [5]. Means were formed for the values determined at various rostrocaudal brain levels. Data were expressed as cell number per mm^2^.

### Analysis of CD31^+^/BrdU^+^ Proliferating Endothelial Cells

The number of CD31^+^/BrdU^+^ endothelial cells was counted in the peri-infarct cortex in every 10th brain section by evaluating regions of interest measuring 0.7386 mm^2^ [2, 4]. Means were formed for cell numbers determined at different rostrocaudal brain levels. Cell counting was done by two independent observers, of which mean values were formed. Data were expressed as cell number per mm^2^.

### Statistical Analysis

Longitudinal data (body weight, behavioral tests) were evaluated by repeated measurement ANOVA followed by Dunnett’s multiple comparisons tests. Cross-sectional histochemical data were assessed by one-way ANOVA followed by Tukey’s multiple comparisons tests. Data are mean ± SEM or SD values. *p* values < 0.05 were considered to indicate statistical significance. For statistical analysis, GraphPad software was used.

## Results

### Hypoxic MSC-sEV Administration Nominally, but Not Statistically Significantly Attenuates Post-ischemic Body Weight Loss

Following MCAO, food intake may pose difficulty for rats exhibiting stroke-related motor and swallowing impairments. Therefore, body weight is a surrogate marker of stroke severity in rats [4]. In this study, we exposed male Sprague–Dawley rats to permanent distal MCAO. Four groups of rats were evaluated, which received treatment with vehicle, platelet-sEVs or MSC-sEVs (2 × 10^6^ or 2 × 10^7^ MSC equivalents/kg) (10 rats/group). Rats revealed a mild loss of body weight of ~ 5% of baseline in the first week post-MCAO (Fig. 1B). The weight loss was nominally lower in rats treated with hypoxic MSC-sEVs at both doses (2 × 10^6^ or 2 × 10^7^ MSC equivalents/kg) or in rats receiving platelet sEVs than in rats receiving vehicle. In the following weeks, body weight almost completely recovered to baseline in all groups (Fig. 1B).

### Hypoxic MSC-sEV Delivery Enhances Post-ischemic Motor-Coordination Recovery

To evaluate the effects of hypoxic MSC-sEVs on motor-coordination impairments, rotating pole and cylinder tests were used. At 24 h after MCAO, all rats exhibited markedly reduced spontaneous motor activity, making it impossible to examine their fine motor skills. For this reason, behavioral studies were started 3 days post-MCAO (that is, 2 days after the initiation of treatment). In this study, motor-coordination deficits partly improved over 28 days post-MCAO, which is in line with earlier studies in younger rats exposed to this distal MCAO model [4]. MSC-EVs significantly enhanced motor-coordination impairments post-MCAO, as outlined in the following.

**Fig. 2:**
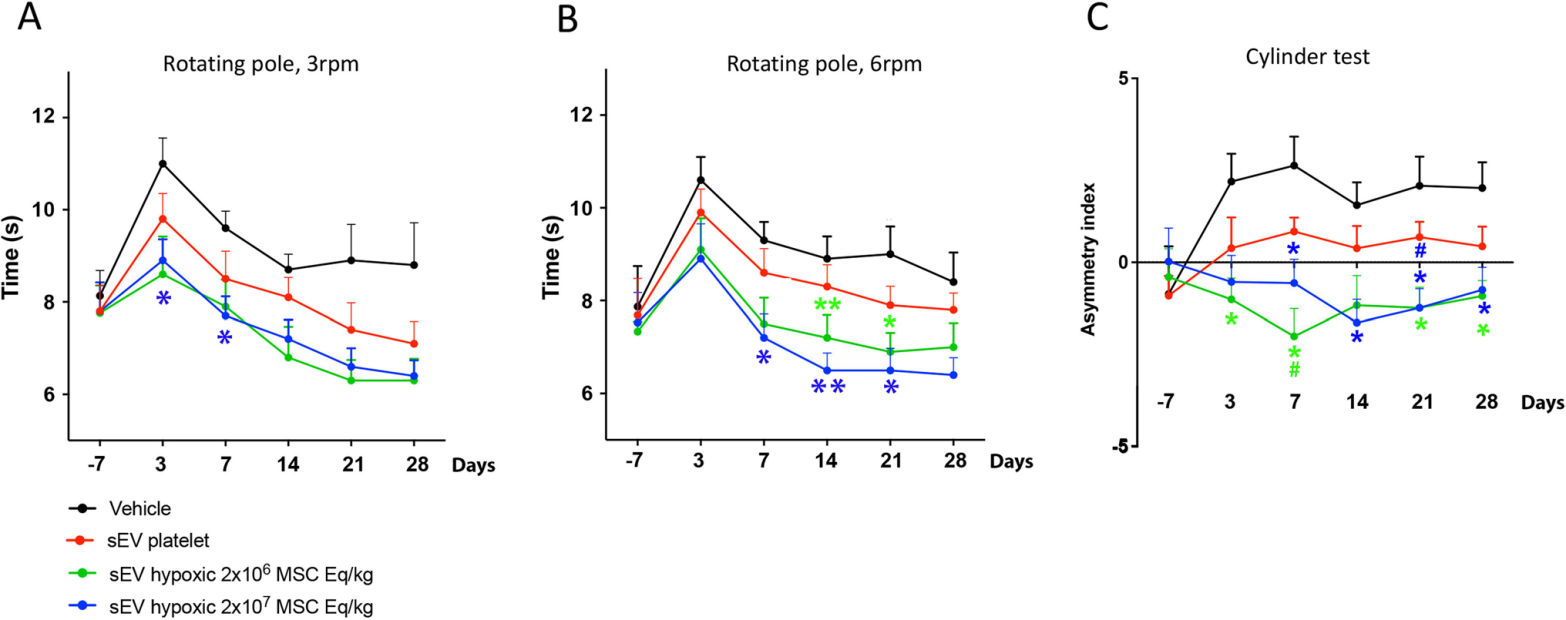
Hypoxic MSC-sEV delivery enhances post-ischemic motor-coordination recovery. Motor-coordination deficits in **A** the rotating pole test at 3 rpm, **B** the rotating pole test at 6 rpm, and **C** the cylinder test performance of rats exposed to permanent distal MCAO, which were intravenously treated with vehicle, platelet-derived sEVs or sEVs obtained from hypoxic MSCs (1% O_2_; 2 × 10^6^ or 2 × 10^7^ cell equivalents/kg) at 1, 3, 7, and 14 days post-MCAO (*n* = 10 animals per group). The rotating pole test measures the duration required to cross a pole rotating at a defined speed of 3 or 6 rpm and the cylinder test evaluates asymmetry in forelimb usage, with more negative results indicating a decreased use of the left affected limb. Note that hypoxic MSC-sEV delivery improved motor coordination recovery in rats exposed to permanent distal MCAO at both doses. *p < 0.05/**p < 0.01 compared with vehicle; ^#^*p* < 0.05 compared with platelet-sEVs. Data are shown as mean ± SEM values

- *Rotating pole test (3 rpm).* Significant deficits in motor-coordination, evidenced by a prolongation of the time needed to traverse the rotating pole, were noted in rats exposed to MCAO (Fig. 2A, B). These deficits were larger in rats evaluated on the pole at 6 rpm than 3 rpm, in line with a higher difficulty of the test. Repeated measurement ANOVA revealed a significant main effect of treatment (*p* = 0.0061) and time (*p* < 0.0001) on rat performance in the rotating pole test at 3 rpm (Fig. 2A). In post hoc analyses, administration of hypoxic MSC-sEVs at a dose of 2 × 10^7^ MSC equivalents/kg significantly enhanced rotating pole deficits at 3 days (*p* = 0.0433) and 7 days (*p* = 0.0164) post-MCAO compared to vehicle (Fig. 2A). Motor-coordination performance spontaneously improved within 7–28 days after MCAO in all groups.
- *Rotating pole test (6 rpm).* In line with the higher test difficulty, motor-coordination deficits in the rotating pole test were more reproducible at 6 rpm than 3 rpm. Repeated measurement ANOVA again showed a significant main effect of treatment (*p* = 0.0038) and time (*p* < 0.0001) on rotating pole performance at 6 rpm (Fig. 2B). In post hoc analyses, MSC-sEV delivery at a dose of 2 × 10^7^ MSC equivalents/kg significantly improved rotating pole deficits at 7 days (*p* = 0.0225), 14 days (*p* = 0.0053), and 21 days (*p* = 0.0209) post-MCAO compared to vehicle (Fig. 2B). Moreover, MSC-sEV delivery at a dose of 2 × 10^6^ MSC equivalents/kg significantly enhanced rotating pole deficits at 14 days (*p* = 0.0053) and 21 days (*p* = 0.0458) (Fig. 2B). Motor-coordination performance again spontaneously improved within 7–28 days post-MCAO in all groups.
- *Cylinder test.* The cylinder test revealed a preference for the unaffected right forelimb in vehicle-treated rats exposed to MCAO (Fig. 2C). Repeated measurement ANOVA showed a significant main effect of treatment (*p* = 0.0001) and a significant interaction effect of treatment and time (*p* = 0.0272) on rat performance in the cylinder test (Fig. 2C). In post hoc analyses, MSC-sEV administration at a dose of 2 × 10^7^ MSC equivalents/kg significantly improved cylinder test deficits at 7 days (*p* = 0.0272), 14 days (*p* = 0.0102), 21 days (*p* = 0.0148), and 28 days (*p* = 0.0372) post-MCAO compared to vehicle and at 21 days (*p* = 0.0463) compared to platelet sEVs (Fig. 2C). Moreover, MSC-sEV administration at a dose of 2 × 10^6^ MSC equivalents/kg significantly enhanced cylinder test deficits at 3 days (*p* = 0.0177), 7 days (*p* = 0.0024), 21 days (*p* = 0.0166), and 28 days (*p* = 0.0128) post-MCAO compared to vehicle, and at 7 days (*p* = 0.0222) compared to platelet sEVs (Fig. 2C). Cylinder test performance only modesty improved over time in vehicle-treated rats in this study.

### Hypoxic MSC-sEVs Do Not Influence Brain Infarct Volume

**Fig. 3:**
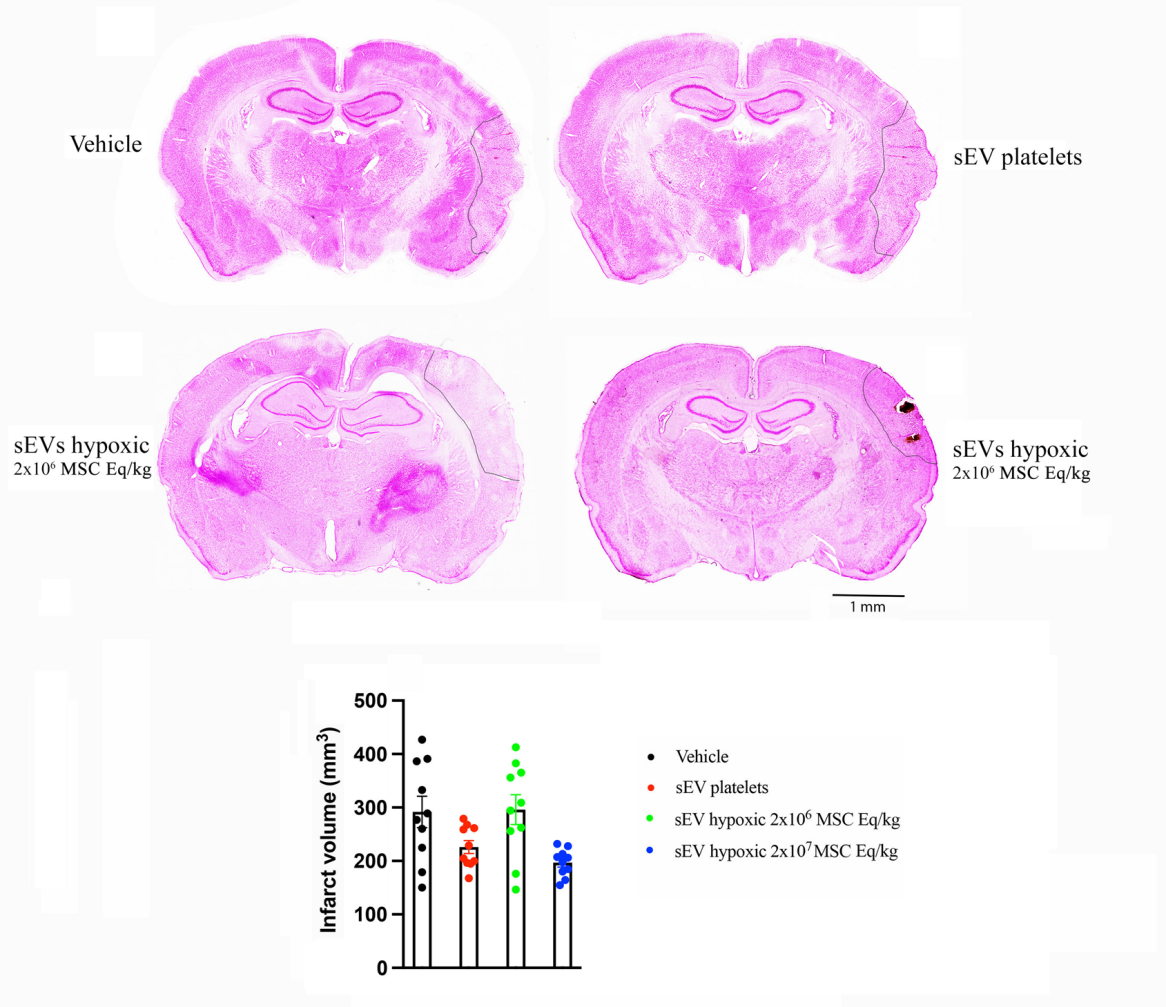
Hypoxic MSC-sEVs do not influence infarct volume after permanent distal MCAO. Infarct volume assessed by methyl green/pyronine Y staining in the brains of permanent distal MCAO rats, which obtained vehicle, platelet-derived sEVs or sEVs obtained from hypoxic MSCs (2 × 10^6^ or 2 × 10^7^ cell equivalents/kg) at 1, 3, 7, and 14 days post-MCAO and which were sacrificed at 28 days post-MCAO (that is, 27 days post-treatment onset). Representative microphotographs are also shown. Note that reproducible brain infarcts covering the lateral parietal cortex were obtained. Infarct volume was not influenced by MSC-sEV delivery at both doses (2 × 10^6^ or 2 × 10^7^ MSC equivalents/kg). Data did not statistically differ between groups. Data are mean ± SEM values. Data of individual animals are depicted as dots. Scale bar, 1 mm

To evaluate effects of MSC-sEVs on long-term brain tissue survival, infarct volume was evaluated by methyl green/pyronine Y stainings at 28 days post-MCAO. Reproducible brain infarcts were observed covering the lateral parietal cortex (Fig. 3). MSC-sEVs at both doses (2 × 10^6^ or 2 × 10^7^ MSC equivalents/kg) did not influence infarct volume to significant extent (Fig. 3).

### Hypoxic MSC-sEVs Decrease Peri-infarct Brain Macrophage and Microglia Accumulation

**Fig. 4:**
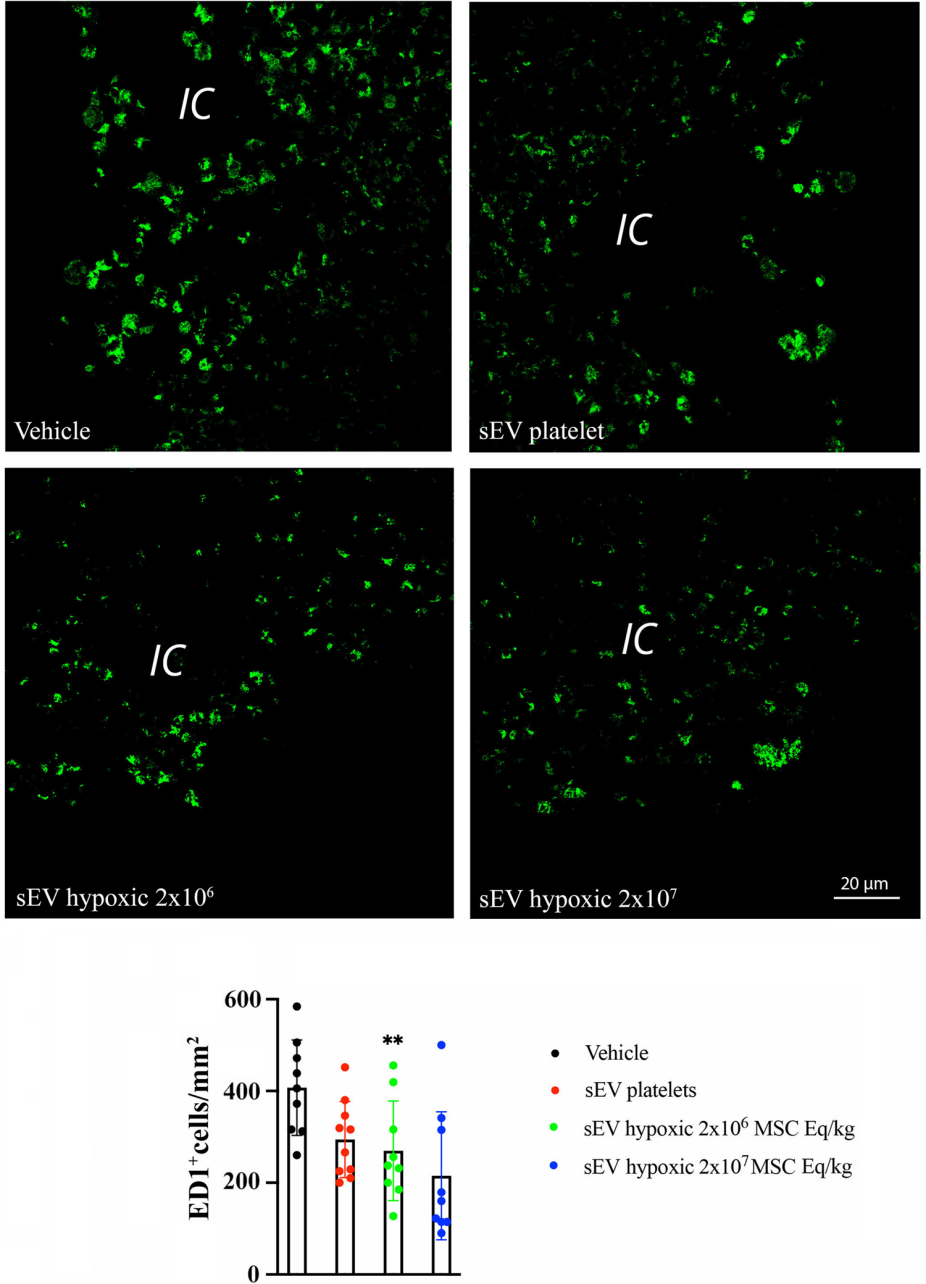
Hypoxic MSC-sEVs decrease peri-infarct brain macrophage infiltrates. Density of activated ED1^+^ macrophages in the peri-infarct tissue of rats, which were treated with vehicle, platelet-derived sEVs or sEVs obtained from hypoxic MSCs (2 × 10^6^ or 2 × 10^7^ cell equivalents/kg) at 1, 3, 7, and 14 days post-MCAO and which were sacrificed at 28 days post-MCAO. Macrophages were discriminated from monocytes through their size, which is ~ 50% larger than that of monocytes. Hence, monocytes were excluded from cell countings. Representative microphotographs are depicted. Note that the MSC-sEVs at a dose of 2 × 10^7^ MSC equivalents/kg reduce peri-infarct ED1^+^ macrophage infiltrates. IC, ischemic cortex. **p < 0.01 compared with vehicle. Data are mean ± SD values. Data of individual animals are depicted as dots. Scale bar, 20 µm

**Fig. 5:**
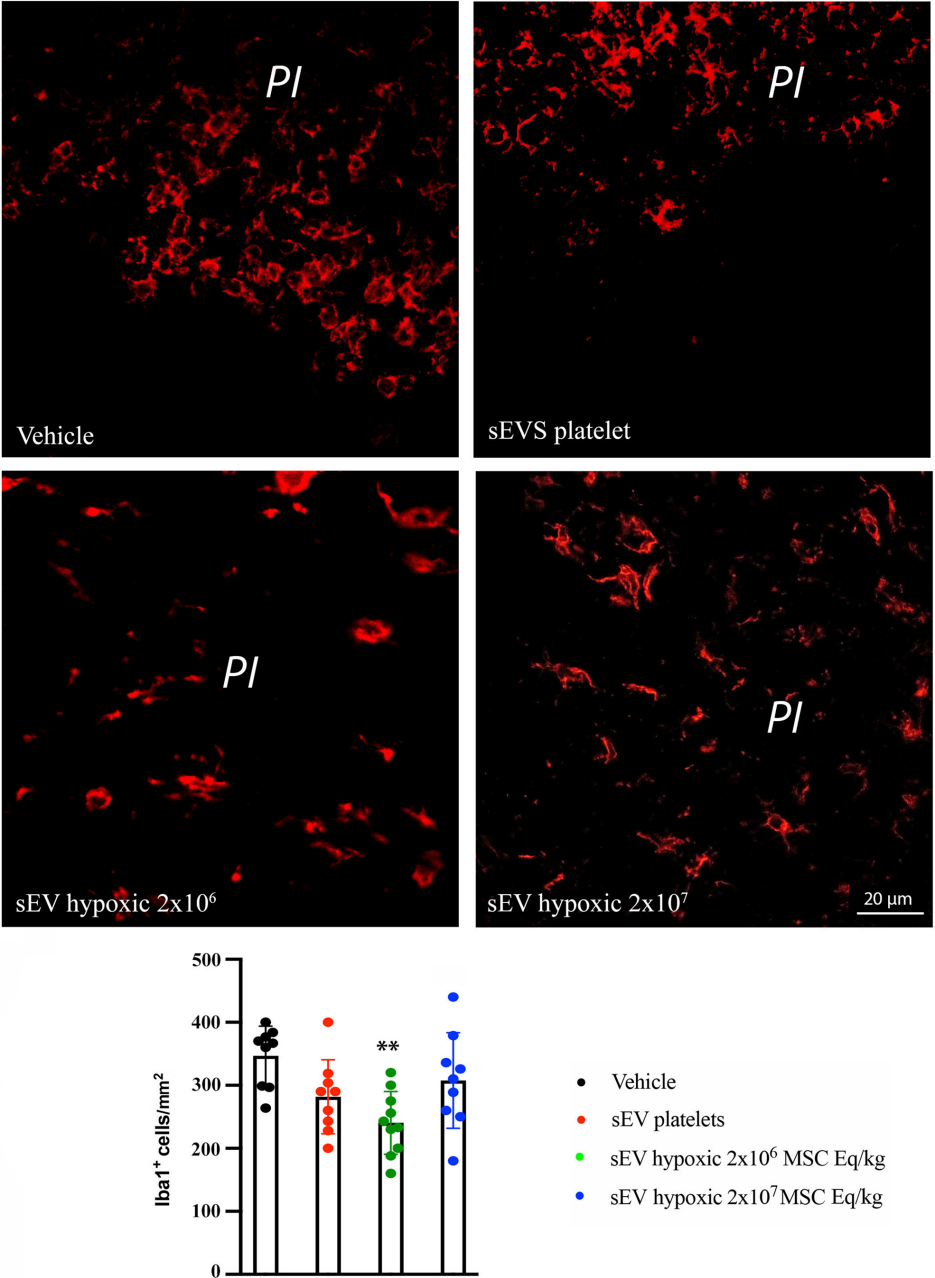
Hypoxic MSC-sEVs decrease peri-infarct microglia accumulation. Density of Iba1^+^ microglia in the peri-infarct brain tissue of rats, which were treated with vehicle, platelet-derived sEVs or sEVs obtained from hypoxic MSCs (2 × 10^6^ or 2 × 10^7^ cell equivalents/kg) at 1, 3, 7, and 14 days post-MCAO and which were sacrificed at 28 days post-MCAO. Representative microphotographs are shown. Note that the MSC-sEVs at a dose of 2 × 10^6^ MSC equivalents/kg reduce peri-infarct Iba1^+^ microglia accumulation. PI, peri-infarct cortex. **p < 0.01 compared with vehicle. Data are mean ± SD values. Data of individual animals are depicted as dots. Scale bar, 20 µm

Ischemic stroke triggers an inflammatory response hindering successful brain tissue remodeling. Brain-infiltrating macrophages and microglial cells are part of this response [4, 23]. We therefore asked if activated macrophage and microglia accumulation was attenuated by hypoxic MSC-sEV administration. At 28 days post-MCAO, a significant number of ED1^+^ activated macrophages, which were distinguished from monocytes based on their size characteristics, and of Iba1^+^ microglia was observed in the peri-infarct brain tissue of vehicle-treated mice (Figs. 4 and 5). Hypoxic sEVs at a dose of 2 × 10^7^ MSC equivalents/kg significantly reduced the peri-infarct ED1^+^ macrophage infiltrates, when compared to vehicle (*p* = 0.0076) (Fig. 4). Similarly, hypoxic sEVs at a dose of 2 × 10^6^ MSC equivalents/kg significantly reduced the peri-infarct Iba1^+^ microglia numbers (*p* = 0.0073) (Fig. 5). Taken together, these data revealed a robust anti-inflammatory effect of hypoxic MSC-sEVs.

### Hypoxic MSC-sEVs Enhance Cerebral Angiogenesis in the Peri-infarct Cortex

**Fig. 6:**
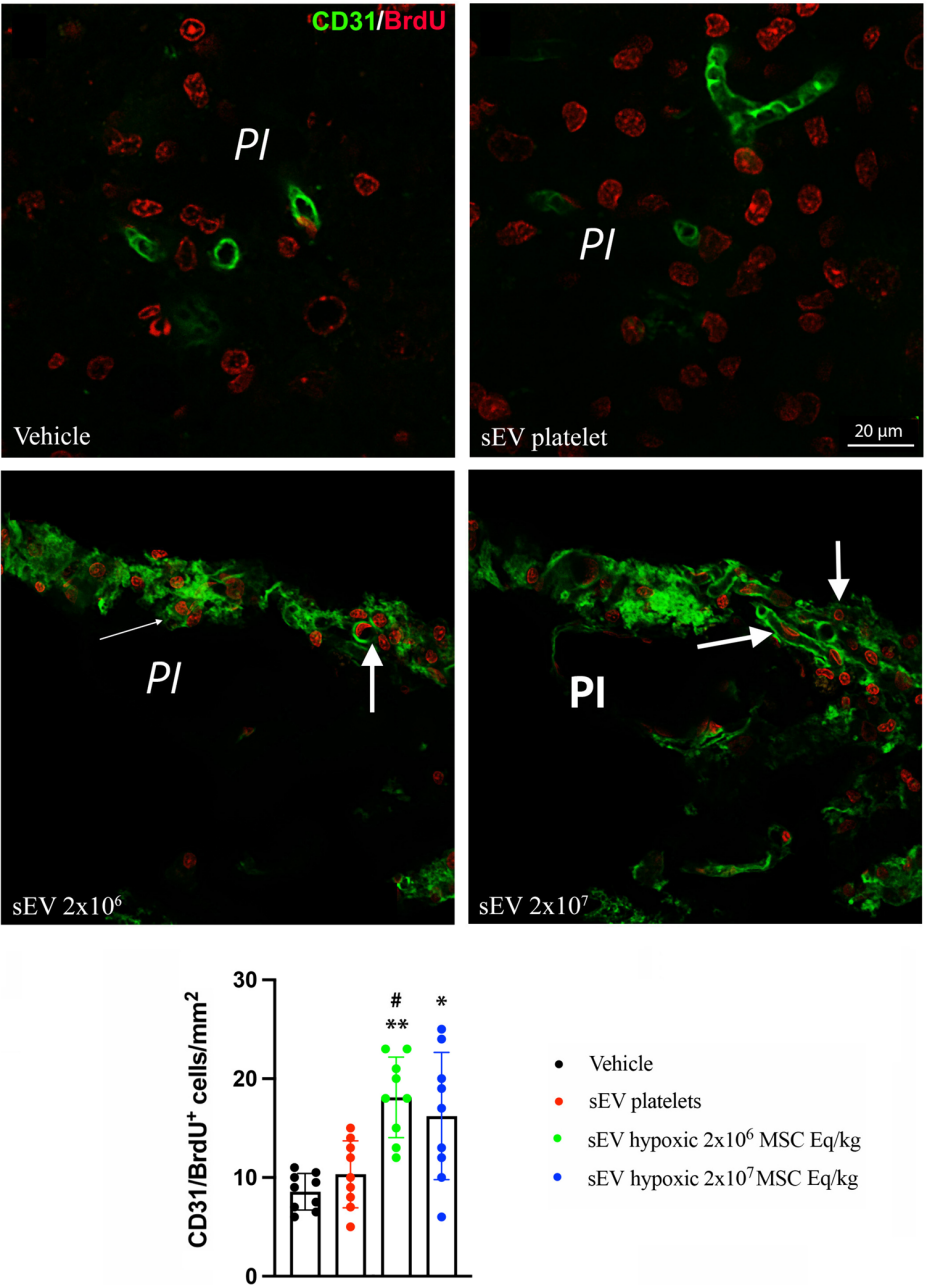
Hypoxic MSC-sEVs enhance post-ischemic angiogenesis in the peri-infarct cortex. Density of CD31^+^/BrdU^+^ proliferating endothelial cells in the peri-infarct cortex of rats, which were treated with vehicle, platelet-derived sEVs or hypoxic MSC-sEVs (2 × 10^6^ or 2 × 10^7^ cell equivalents/kg) at 1, 3, 7, and 14 days post-MCAO, followed by animal sacrifice at 28 days post-MCAO. For proliferating endothelial cell labeling, BrdU (50 mg/kg) was intraperitoneally administered from 8 to 18 days post-MCAO. Representative microphotographs are shown. Arrows depict double-labeled cells. Note that MSC-sEVs at doses of 2 × 10^6^ and 2 × 10^7^ MSC equivalents/kg increase endothelial proliferation in the peri-infarct cortex. PI, peri-infarct cortex. *p < 0.05/**p < 0.01 compared with vehicle/^#^*p* < 0.05 compared with platelet-sEVs. Data are mean ± SD values. Data of individual animals are depicted as dots. Scale bar, 20 µm

In a previous study, we found that sEVs obtained from hypoxic, but not normoxic MSCs potently increased peri-infarct microvascular remodeling and angiogenesis in mice exposed to transient proximal MCAO [7]. We therefore asked if hypoxic MSC-sEVs also promoted angiogenesis in the peri-infarct cortex of rats exposed to permanent distal MCAO. A moderate number of CD31^+^/BrdU^+^ proliferating endothelial cells was found in the peri-infarct cortex of rats receiving vehicle or platelet-sEVs at 28 days post-MCAO (Fig. 6). Administration of hypoxic MSC-sEVs at both doses (2 × 10^6^ or 2 × 10^7^ MSC equivalents/kg) significantly increased the number of CD31^+^/BrdU^+^ endothelial cells by 1.8–2.0-fold, when compared to vehicle and to platelet-sEVs (*p* < 0.001 for all comparisons) (Fig. 6). These observations confirm a pro-angiogenic action of hypoxic MSC-sEVs in the rat permanent distal MCAO model.

## Discussion

In a rat permanent distal MCAO model, we provide evidence that sEVs obtained from hypoxic MSCs (1% O_2_) promote neurological recovery and brain remodeling, when administered in the post-acute stroke phase starting 24 h after MCAO. In this study, two different sEV doses were examined (2 × 10^6^ or 2 × 10^7^ MSC equivalents/kg), which were injected four times post-MCAO (at 24 h, 3, 7, and 14 days). Hypoxic MSC-sEVs at both doses improved motor-coordination recovery, as examined by a battery of rotating rod and cylinder tests. The neurological improvements persisted throughout the follow-up of 28 days. Infarct volume was not influenced in this permanent MCAO model by hypoxic MSC-sEVs. The absence of infarct volume changes is in line with previous studies of our group in rats exposed to permanent distal MCAO [4] and mice exposed to transient proximal MCAO [3], in which the delivery of normoxic MSC-sEVs (harvested at 21% O_2_) also did not influence infarct size after 28 days, when administered starting 24 h post-injury. Brain infarcts rapidly expand in the first 24 h post-MCAO [11, 14]. At this time-point, brain infarcts have achieved their final size in permanent MCAO models [8, 14]. It is therefore not surprising that MSC-sEV treatment initiated after 24 h did not alter infarct volume in this study.

The observation that sEVs obtained from hypoxic MSCs promote motor-coordination recovery, when administered in the post-acute stroke phase, is new. We previously reported that in a model of transient proximal MCAO in mice that the delivery of sEVs obtained from hypoxic MSCs (also harvested at 1% O_2_), but not sEVs obtained from normoxic MSCs (harvested at 21% O_2_) increased long-term neuronal survival and reduced brain atrophy after 56 days, when initiated at the same time-point, i.e., 24 h post-MCAO [7]. In this earlier study, we did not evaluate motor-coordination recovery using dedicated sensorimotor tests. Of note, in the present study, functional neurological improvements were similarly observed at a dosage 10 times lower (2 × 10^6^ MSC equivalents/kg) to that previously tested (2 × 10^7^ MSC equivalents/kg) [7]. This finding underlines the potency of hypoxic MSC-sEVs. That hypoxic MSC-sEVs reduced the peri-infarct accumulation of activated ED1^+^ macrophages and Iba1^+^ microglia is in line with earlier studies of our group for normoxic MSC-sEVs, which decreased activated ED1^+^ macrophages and Iba1^+^ microglia in the peri-infarct brain tissue, when administered starting 24 h after permanent distal MCAO in rats [4]. This observation is also in line with our previous findings for hypoxic MSC-sEVs, which reduced CD11b^+^ CD62L^+^ macrophage infiltrates in ischemic brain tissue, when administered immediately after transient proximal MCAO in mice [22]. The anti-inflammatory effect of hypoxic MSC-sEVs may have contributed to a microenvironment favorable for brain tissue remodeling.

In line with earlier mouse studies examining the efficacy of hypoxic MSC-sEVs [7, 23], the present study found that hypoxic MSC-sEVs enhanced peri-infarct angiogenesis evaluated by BrdU incorporation analysis in rats at both sEV dosages. In transient proximal MCAO mice, we previously described that sEVs obtained from normoxic MSCs (harvested at 21% O_2_) and hypoxic MSCs (harvested at 1% O_2_) similarly reduced ischemic injury and brain leukocyte infiltrates [22, 23]. In contrast to normoxic MSC-sEVs, hypoxic MSC-sEVs also increased blood–brain barrier integrity and peri-infarct angiogenesis [7, 23]. In this earlier study, we performed a broad set of cell culture studies, in which we administered normoxic and hypoxic MSC-sEVs obtained from various human donors to human cerebral microvascular endothelial hCMEC/D3 cells. sEVs obtained from hypoxic MSCs (harvested at 1% O_2_), but not normoxic MSCs (harvested at 21% O_2_) consistently enhanced angiogenesis, as revealed by endothelial proliferation, migration, and tube formation assays [7]. The present study examined male MCAO rats. We previously evaluated the efficacy of sEVs derived from hypoxic MSCs in male and female mice post-MCAO [22]. In that earlier study, therapeutic effects were equally noted in both sexes.

In our previous studies, proteome analyses of MSC-sEVs isolated under hypoxic and normoxic conditions revealed that proteins with restorative functions involved in extracellular matrix–receptor interaction, focal adhesion, and cell migration were enriched in hypoxic MSC-sEVs, whereas proteins with opposite functions impeding restorative processes and cell metabolism were reduced [7]. Whether hypoxia-inducible factor-1α (HIF-1α) plays a central role in modulating the composition of hypoxic MSC-sEVs, as indicated by an earlier study on human umbilical vein endothelial cells (HUVECs) [6], remains to be shown. In the latter study, MSCs overexpressing HIF-1α were found to exhibit an increased abundance of the Notch ligand Jagged-1 in sEVs [6]. Jagged-1-positive sEVs were found to stimulate angiogenesis in tube formation and plug assays [6]. The angiogenic effects of Jagged-1-positive sEVs were blocked by sEV incubation with an anti-Jagged-1 antibody.

Taken together, we found that sEVs obtained from hypoxic MSCs improve motor-coordination recovery in rats exposed to permanent distal MCAO, reduce peri-infarct activated macrophage and microglia accumulation, and enhance peri-infarct angiogenesis. This study supports the notion that hypoxic conditioning might represent a promising strategy for obtaining MSC-sEVs for clinical stroke studies.

## Supplementary Information

Below is the link to the electronic supplementary material.

Supplementary file1 (DOCX 110 KB)

## Data Availability

Data is provided with the manuscript or supplementary information files.
