# Supplementary material for: Extracellular Vesicles Obtained from Hypoxic Mesenchymal Stromal Cells Induce Neurological Recovery, Anti-inflammation, and Brain Remodeling After Distal Middle Cerebral Artery Occlusion in Rats
Source: Transl Stroke Res. 2024 Sep 7;16(3):817–30. doi: 10.1007/s12975-024-01266-5 (PMC12045817; doi:10.1007/s12975-024-01266-5)

**Supplementary Table 1. Particle concentration, mean particle size, protein concentration and sEV purity of the MSC-sEV preparation assessed by nanoparticle tracking analysis (NTA) and bicinchoninic acid (BCA) assay.**

| Preparation | Particle concentration [particles/ ml] | Particle size [nm] | Protein concentration [µg/ µl] | Purity [particles/ mg protein] |
| --- | --- | --- | --- | --- |
| Hypoxic MSC-sEVs | 2.8*10^11^ | 121.9 | 2.41 | 1.2*10^11^ |

**Supplementary Table 2. Concentration of CD9^+^, CD63^+^ and CD81^+^ particles assessed by imaging flow cytometry.**

| Preparation | CD9 [objects/ml] | CD63  [objects/ml] | CD81  [objects/ml] |
| --- | --- | --- | --- |
| Hypoxic MSC-sEVs | 1.16*10^9^ | 2.03*10^8^ | 3.01*10^8^ |

**Supplementary Table 3. Antibodies used for imaging flow cytometry.**

| Antibody | Company | Clone |
| --- | --- | --- |
| CD9 PE | EXBIO | MEM-61 |
| CD63 APC | EXBIO | MEM-259 |
| CD81 FITC | Beckmann Coulter | JS-64 |

**Supplementary Figure 1. Gating strategy used for imaging flow cytometry.**


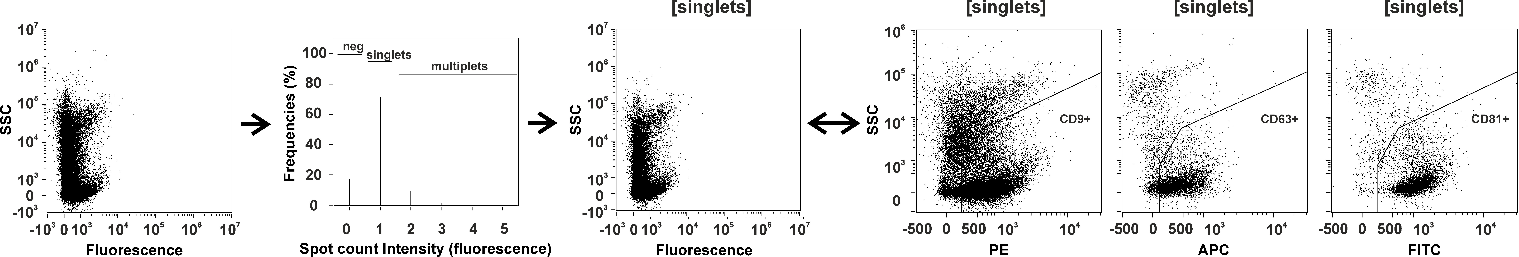

Supplement: Supplementary file 1 — Supplementary file1 (DOCX 110 KB) [file 12975_2024_1266_MOESM1_ESM.docx]
